# Transceiver 16‐Channel Coaxial‐End Dipole Array for Combined Head and C‐Spine MRI at 9.4 T

**DOI:** 10.1002/nbm.70228

**Published:** 2026-01-26

**Authors:** G. A. Solomakha, F. Glang, M. W. May, S. Mueller, J. Walzog, A. Aghaeifar, D. Bosch, J. Bause, O. Kraff, K. Scheffler, H. H. Quick, N. I. Avdievich

**Affiliations:** ^1^ High‐Field MR Center Max Planck Institute for Biological Cybernetics Tübingen Germany; ^2^ Institute of Biomedical Imaging Graz University of Technology Graz Austria; ^3^ Erwin L. Hahn Institute for MRI University Duisburg‐Essen Essen Germany; ^4^ High‐Field and Hybrid MR Imaging University Hospital Essen Essen Germany; ^5^ Electronical Workshop Max Planck Institute for Biological Cybernetics Tübingen Germany; ^6^ Department for Biomedical Magnetic Resonance University of Tübingen Tübingen Germany; ^7^ Core Facility for Magnetic Resonance Imaging, Medical Faculty University of Tübingen Tübingen Germany

## Abstract

This work aims to design a double‐row transceiver array consisting of 16 folded‐end coaxial‐end dipoles for combined C‐spine and brain imaging. The curved coaxial‐end dipole elements were aligned on a tight‐fitting, ergonomically shaped conformal holder optimized for subject comfort. Transmit efficiency, B_1_^+^ field homogeneity, and tissue‐specific absorption rate (SAR) were numerically evaluated using electromagnetic simulations and optimized with respect to several configurations of the array geometry. After identifying the optimal array configuration, the array was built and tested on a bench and in the MRI scanner, using anatomical imaging and transmit field mapping on a phantom and healthy volunteers. The designed array provided RF excitation over the entire brain and cervical spinal cord down to the C7 region, covering a field of view of more than 365 mm in head‐foot direction. Measured in a region covering the cerebrum, cerebellum, brainstem, and C‐spine, it achieved B_1_^+^ field homogeneity of ~31% (coefficient of variation), mean transmit efficiency of ~0.38 μT/√W, and SAR efficiency of 0.65 μT/√W/kg when driven in the circularly polarized mode. The acquired anatomical MR images confirmed that the constructed array provided coverage of the brain and C‐spine.

## Graphical abstract

A double‐row transceiver array consisting of 16 folded‐end coaxial‐end dipoles was designed. Curved dipole elements were aligned on a tight‐fitting, ergonomically shaped holder optimized for subject comfort. Transmit efficiency, B_1_^+^ field homogeneity, and SAR were numerically evaluated using EM‐simulations and optimized with respect to several configurations of the array geometry. After identifying the optimal configuration, the array was built and tested on a bench and in MR‐scanner by anatomical imaging and transmit field mapping on phantom and healthy volunteers.

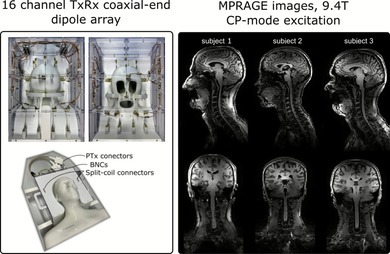

**Abbreviations**
CAD: computer‐aided design
COV: coefficient of variation
pTx: parallel transmit
RF: radiofrequency
ROI: region of interest
SAR: specific absorption rate
SNR: signal‐to‐noise ratio
ToRo: transmit only/receive only
TxRx: transmit/receive
UHF: ultrahigh fields
VOPs: virtual observation points

## 1 Introduction

Magnetic resonance imaging (MRI) at ultrahigh fields (UHF, static magnetic field B_0_ ≥ 7 T) can provide increased signal‐to‐noise ratio (SNR) and contrast‐to‐noise ratio compared to MRI at lower fields [1, 2, 3]. Currently, there are more than 100 UHF MRI systems for human use worldwide, of which the most powerful with B_0_ > 7 T have 9.4, 10.5, and 11.7 T [4]. In general, MRI applications that require high imaging speed or resolution can benefit from UHF. One of such applications is combined imaging of the brain and the cervical spine (C‐spine). Using UHF for this task promises more detailed insights into various pathologies, such as multiple sclerosis or similar neuroinflammatory diseases [5] and Alzheimer's disease [6]. This perspective has become even more relevant with the recent clinical approval of UHF systems [7]. In addition, BOLD functional MRI studies of the central nervous system can benefit from UHF because of the increased sensitivity to subtle functional signal changes and better specificity to small structures like gray matter within the spinal cord [8, 9, 10].

Simultaneous imaging of the brain and C‐spine at UHF requires a local transmit (Tx) coil providing an excitation over the entire area (~35 cm in head–foot direction). However, practical development of RF coils covering such a large volume is a very challenging task because of the strong inhomogeneity of the RF magnetic field (usually referred to as B_1_^+^) and local RF tissue heating [11] (an increase in the specific absorption rate [SAR]). RF field inhomogeneities are caused by substantial shortening of the wavelength compared to 1.5 and 3 T (close to 10 cm at 400 MHz) in human body tissues because of their large permittivity [12] (below 60 for human brain tissues at 400 MHz). The B_1_^+^ inhomogeneity problem can be partially mitigated by using multielement Tx arrays, which were introduced first for 4 T [13] and later adapted for 7 T [14, 15, 16] and 9.4 T [17, 18, 19] brain MR imaging, possibly in conjunction with parallel transmission (pTx) in the form of static RF shimming [20, 21] or dynamic pTx pulses [22, 23, 24]. The number of elements in such an array is usually limited by the number of channels in the pTx system. A typical RF coil setup consists of eight [25] or 16 loops [17] as Tx elements with up to 96 [26] and even 128 [27] additional receive (Rx)‐only loops. Depending on the design, Tx elements could be used only during transmission (so‐called Transmit‐only/Receive‐only array [ToRo] [17]) or both in transmission and reception (so‐called transceiver [TxRx] array) [15, 16, 26, 27]. In addition, 3D RF shimming requires using multirow Tx arrays, e.g., two rows of eight loops (2 × 8 head arrays) [21, 28, 29]. However, due to difficulties of B_1_^+^ shimming over the entire brain/C‐spine area at UHF, most of the current 7 T RF coil developments have targeted either the brain [17, 26, 29, 30, 31, 32, 33] or C‐spine [34, 35, 36], using both the ToRo and TxRx approaches, usually based on loop elements [34, 36, 37, 38]. To the best of our knowledge, there are no reported studies of combined brain/C‐spine imaging at ≥ 9.4 T and only one study of C‐spine imaging [39]. Recently, some works attempted to extend the Tx coverage over the brain and C‐spine (or partial C‐spine) using different 7 T array designs and mainly loops. This included combining a commercial [40] or home‐built brain‐only array [41] with several Tx loops positioned in the neck area or designing a long 2 × 8 loop array covering both head and neck [42, 43]. However, at UHF, such loop arrays have several disadvantages. First, long loops require many high‐voltage and low‐loss capacitors to be distributed along the loop conductors, e.g., 16 capacitors per loop at 300 MHz [41] and even more at 400 MHz. Still, in many cases, the values of these capacitors do not substantially exceed the parasitic capacitance (i.e., up to several pF) between the opposite conductors (opposite sides of rectangle/square) of the loop, which makes the current distribution along the loop conductors nonuniform and, therefore, the loop's performance suboptimal. Another disadvantage of the loop‐only arrays is the suboptimal receive performance (SNR) in the central brain region. According to the ultimate intrinsic SNR (UISNR) theory [44, 45], at UHF, to achieve the maximum possible SNR near the center of the human brain, dipole‐like elements should be combined with loops. This was also experimentally confirmed by several studies at both 7 T [29, 31, 46] and 9.4 T [30, 47, 48].

Dipoles, which were introduced about 10 years ago for human body imaging at 7 T [49], are a compelling alternative to loop elements of an array. Later, dipoles were adapted for C‐spine imaging [35] and for brain imaging at 7 T [29, 31, 46, 50, 51], 9.4 T [48, 52, 53, 54, 55, 56], and even higher field strengths [57, 58]. Dipoles are much simpler to construct than loops, since no distributed capacitors are required. Also, dipoles have been shown to improve the longitudinal (along the main magnetic field) Tx coverage compared to loop arrays at 9.4 T [53, 59] and outperform commercial state‐of‐the‐art arrays at 7 T [31, 51]. Since at UHF, dipoles can also improve central SNR [29, 31, 46, 48], it is beneficial to use them both in transmission and reception, i.e., in a tight‐fit TxRx configuration. In addition, combining TxRx dipoles with Rx‐only loops helps minimize the total number of array elements [31] and, hence, simplifies coil design. However, as demonstrated previously at both 7 T [48] and 9.4 T [48], high voltage (electric field) at the ends of common dipole antennas positioned very close to the sample, as found in tight‐fit dipole TxRx arrays, may produce a substantial shift of the resonance frequency due to variation in the distance to the sample. This makes the usage of common dipoles in tight‐fit arrays very difficult. To minimize the frequency shift, we recently developed several types of novel dipole antennas. In the folded‐end dipole design [48, 51, 53, 54, 59], this effect was minimized by folding and moving the dipole's ends away sample. In the coaxial and [56] coaxial‐end dipole design [60], which were developed following the work of [61], two short (2 to 4 cm) pieces of a coaxial cable shorted by lumped inductors are placed at the ends of each dipole. Both folded‐end and coaxial (both coaxial and coaxial‐end) dipole antennas improved current distribution along the length of the dipole, decreased local SAR at the surface of the sample, and minimized the frequency shift due to variation in head sizes as compared to the common straight dipoles [51, 53, 54, 56]. At the same time, coaxial dipoles provide a more compact design, deliver better SAR efficiency, and provide an easier way to adjust the resonance frequency by varying the end inductor's value as compared to folded‐end dipoles [56]. In addition, adjacent coaxial dipoles are less coupled to each other due to the absence of long folded‐end parts. Finally, the coaxial‐end dipole design allows further simplification of array elements by replacing the central part of the coaxial cable by a wire [60].

In this work, we present a 16‐channel tight‐fit dual‐row folded‐end coaxial‐end dipole array, which allows for extended coverage over the entire brain and C‐spine at 9.4 T. For this purpose, dipole array elements were placed on a thoroughly designed, ergonomically shaped, conformal holder. Our simulation and experimental results show that this array provides full‐brain and C‐spine coverage (down to the C7 region). To our best knowledge, this is the first demonstration of using a double‐row dipole array for combined brain/C‐spine imaging at UHF. While the present work introduces a coil design for relatively rare 9.4‐T human MRI, such designs can be adapted for 7 T, which is gaining clinical relevance, and may as well be promising candidates for the next generation of scanners operating at > 10 T. Examples of coils first developed at 9.4 T and then adapted for 7 T are included in but not limited to [17, 26, 28, 31, 51, 59].

## 2 Methods

### 2.1 Design of the Array Holder and Numerical Simulations

**FIGURE 1:**
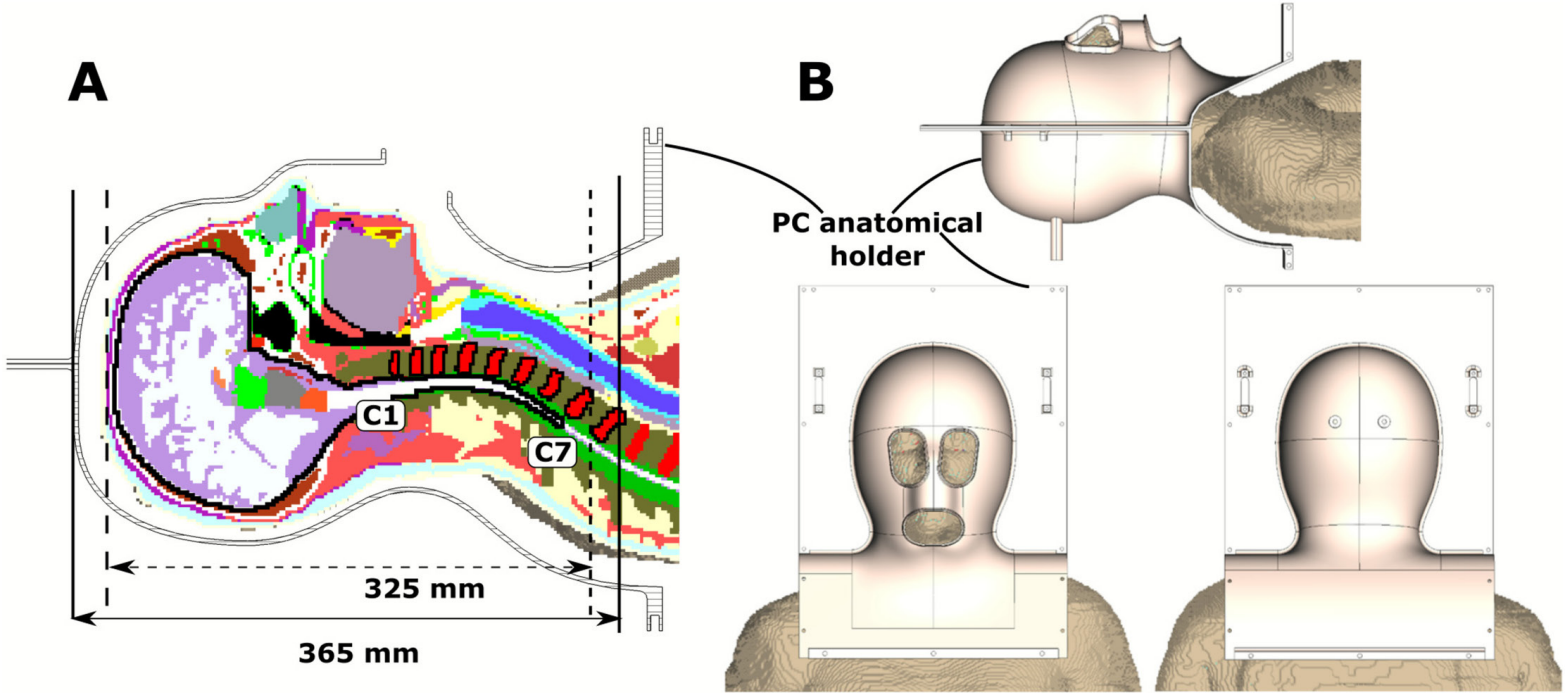
(A) Cross‐section of the polycarbonate anatomical holder for the head and C‐spine coil, and the Duke voxel model. The targeted ROI for evaluating B_1_ fields is shown as a black contour, which includes the entire brain and spinal cord down to C7. (B) Side, top, and bottom view of the anatomical holder with the Duke voxel model.

The ergonomically shaped tight‐fit array holder (coil housing, Figure 1) was designed using Siemens NX CAD software (Siemens DIS, TX, USA). The main criterion for the design of the holder was the subject's comfort within the coil. For this purpose, we performed imaging of the head and upper part of the chest of a healthy male subject with a relatively large head (circumference of 59 cm) and a healthy female subject with a relatively small head (circumference of 54 cm) using an Rx‐only 64‐channel head–neck coil at a Siemens Prisma 3 T (Siemens Healthineers, Germany) MR scanner. MPRAGE [62] images were used to generate a 3D human CAD model, including the chest regions [63]. These models were then imported into the NX CAD software to provide references for the holder design. Since the clinical 3 T coil is used for clinical long‐run scans, we are confident that the obtained geometry can provide a comfortable volunteer position in our coil as well. In addition, to make the design more patient‐friendly, the holder was split into two halves of similar height. The CAD model of the designed anatomical holder was imported to CST Studio 2021 (Dassault Systèmes, France). A sagittal slice through the center of the CAD model of the holder with the Duke multitissue voxel model with 2 mm resolution [64, 65] (Zurich MedTech, Switzerland) is presented in Figure 1A. The model's side, top, and bottom views are presented in Figure 1B. The designed holder had a wall thickness of 3 mm to provide mechanical stability. Polycarbonate (ε = 2.9, tgδ = 0.011) was chosen as the material of choice for the holder because it is 3D printable, low‐loss for RF‐fields, and relatively MR invisible. Based on the anatomy of the Duke voxel model, we estimated that a B_1_^+^ coverage over the 325 mm region would be necessary to cover the entire brain and entire C‐spine (C1‐C7) (see Figure 1A). As demonstrated previously [59], to improve the longitudinal transmit coverage, it is beneficial further to extend the length of the dipoles. Following this approach, we extended the target size region by 40 mm, from 325 to 365 mm (Figure 1A).

**FIGURE 2:**
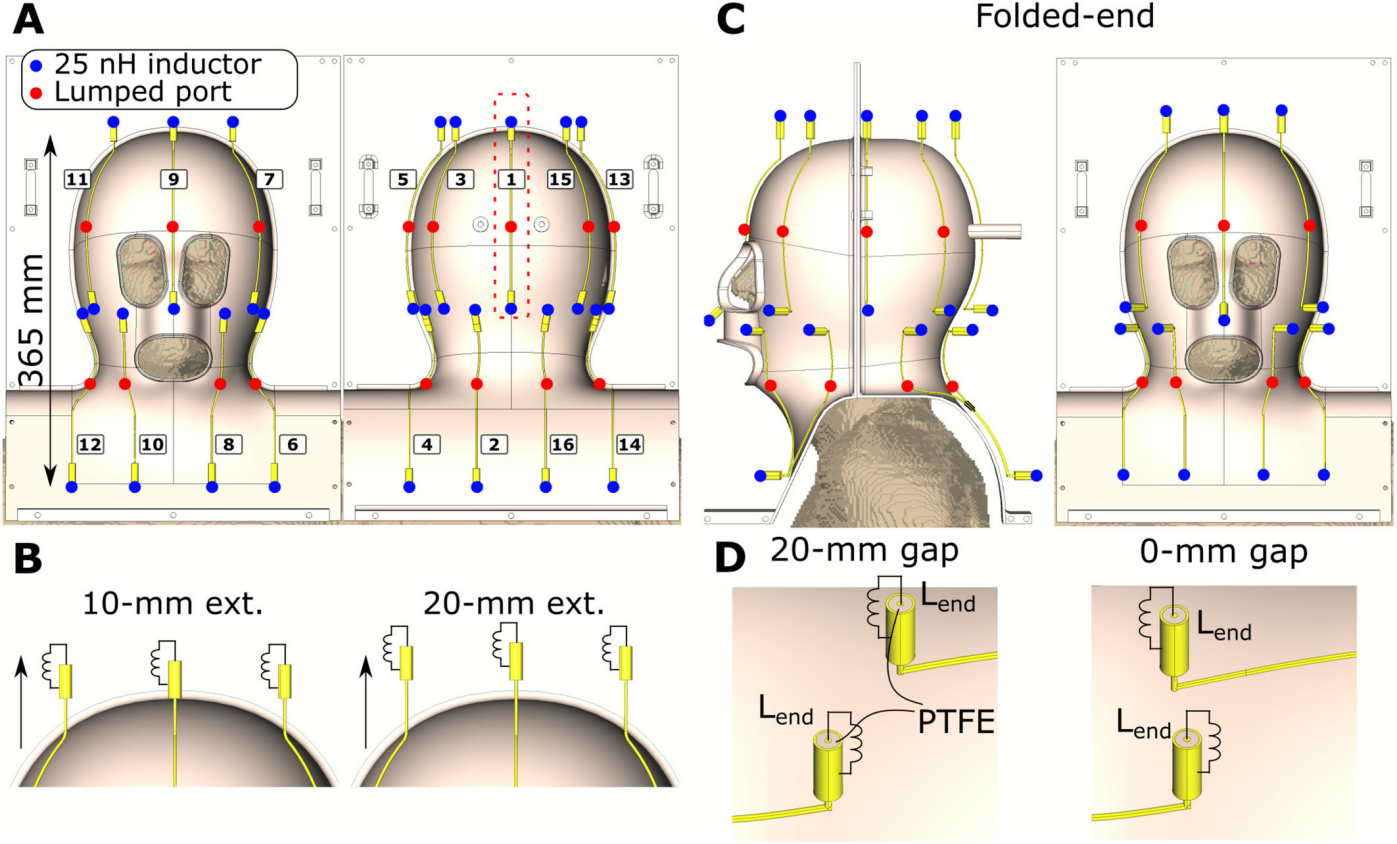
(A) Top and bottom view of the straight (l_1_ = 194 mm) dual‐row 16‐channel coaxial‐end dipole array numerical model. (B) Magnified view of the top part of 10‐mm (l_1_ = 204 mm) and 20‐mm (l_1_ = 214 mm) extended dipole arrays. (C) Side and top view of the folded‐end dual‐row coaxial‐end 16‐channel array with 20‐mm gaps b/w rows (l_1_ = 220 mm). (D) Magnified view of the gaps between the rows for 0‐mm (l_1_ = 240 mm) and 20‐mm (l_1_ = 220 mm) inter‐row gaps.

The 16 coaxial‐end dipoles introduced in work [60] were distributed in two rows to cover the target region, with the length of each row along the z‐axis measuring 182.5 mm, with dipole conductors guided along the holder surface. Top and bottom views of the numerical model of the coaxial‐end dipole array are presented in Figure 2A. Because of the ergonomical shape of the holder, the length of individual dipoles varied depending on the location. Therefore, to compare and distinguish arrays, we chose the length of the element #1, l_1_, located near the back of the head (Figure 2A) as a reference. For the “Straight” array configuration, l_1_ measured 194 mm. The dipoles were constructed using 1.6 mm copper wire. The coaxial ends were 20 mm long for all array elements and made from a 50‐Ohm coaxial cable with an inner conductor diameter of 1.2 mm. Lumped 50 Ohm ports were placed in the middle of each dipole. The shield and center conductors were made from copper, while PTFE (Teflon, ε = 2.1, tgδ = 0.0002) was used as dielectric in the coaxial cables. At the end of each dipole, between the shield and center conduct, located near the back of the head (Figure 2A) as a reference. For the “Straight” array configuration, l_1_ measured 194 mm. The dipoles were constructed using 1.6 mm copper wire. The coaxial ends were 20 mm long for all array elements and made from a 50‐Ohm coaxial cable with an inner conductor diameter of 1.2 mm. Lumped 50 Ohm ports were placed in the middle of each dipole. The shield and center conductors were made from copper, while PTFE (Teflon, ε = 2.1, tgδ = 0.0002) was used as dielectric in the coaxial cables. At the end of each dipole, between the shield and center conductor of the coaxial end, a 25‐nH lumped inductor (L_end_) was placed. The Q‐factor of L_end_ was set to 120. This corresponds to the Q‐factor of a self‐made inductor built from 1.2‐mm copper wire at 400 MHz. This array configuration will be referred to below as “Straight”.

Numerical simulations of all array configurations were performed using the finite integration technique in the time domain (FIT‐TD) implemented in CST Studio 2021. To improve the simulation accuracy, a local mesh with a 0.8 mm step size was applied to all array conductors, resulting in ~80·10^6^ mesh cells. Along with the above‐mentioned Duke multitissue voxel model, Ella from the same model family was used for simulations. We used a high‐performance workstation with four Nvidia Tesla V100 GPUs for simulation. The average simulation time of one model was ~6 h.

All array elements were matched to 50‐Ohm impedance with an L‐matching network consisting of a parallel capacitor and two series inductors (Q = 120) using the CST Studio schematic module. For all considered arrays, matching on the Duke model was adjusted to be better than −30 dB. Simulations on the Ella voxel model were conducted without further adjustments of the matching network. Also, B_1_^+^ was calculated for CP‐mode excitation (−45° phase shift between adjacent elements in the same row and −22.5° between adjacent elements in different rows) for all simulated arrays. To compare different array configurations, we calculated pSAR_10g_ (peak SAR value averaged over 10 g of tissue using the CST Legacy averaging method, evaluated over the entire voxel model), as well as <B_1_^+^_ROI_> (mean B_1_^+^ over an ROI (Figure 1A) that included the cerebrum, cerebellum, brainstem, and spinal cord from C1 to C7). To evaluate homogeneity, the coefficient of variation (COV) of B_1_^+^ was computed in the same ROI. In addition, the SAR efficiency (mean B_1_^+^ over the ROI normalized to √pSAR_10g_) was calculated and compared.

In the next step, we evaluated how an extension of the dipoles above the head affects the array performance. Previous work [59] has demonstrated that such an extension improved B_1_^+^ at the superior location. Two straight coaxial‐dipole array configurations with extended (10 mm [l_1_ = 204 mm] and 20 mm [l_1_ = 214 mm]) dipole length in the top row were evaluated numerically. A schematic view of the extended dipoles is presented in Figure 2B.

Since the ergonomical holder design is relatively tight‐fit, a potentially high pSAR and high sensitivity of the resonance frequency to load variation may still occur [48, 51]. In other studies [51, 59], it was demonstrated that folding the dipole ends and moving them away from the tissue reduced pSAR and decreased the loading effect. Therefore, we also evaluated the change in performance when the ends of the dipoles were folded away from the subject. To create the first configuration of the folded‐end coaxial‐end array, we used the 10‐mm extended configuration (Figure 2A,B) of the straight coaxial‐end array and added the folded parts to the dipoles' ends (except at the top end of the upper row, Figure 2C). This resulted in a folded‐end coaxial‐end array design with a zero gap between the rows (l_1_ = 240 mm) (Figure 2D). Increase in the length of the dipoles in the folded‐end coaxial‐end array inevitably leads to lower matching network inductance values, which in turn may result in suboptimal dipole performance [56]. Therefore, we also evaluated another array configuration with a reduced length of the dipoles in the upper row (20‐mm gap between the rows, l_1_ = 220 mm). The numerical model of the folded‐end coaxial‐end dipoles is presented in Figure 2C. The enclosure views of the dipoles with 0‐ and 20‐mm gaps between the rows are shown in Figure 2D.

All five array configurations, i.e., three straight (0‐mm [l_1_ = 194 mm], 10‐mm [l_1_ = 204 mm], 20‐mm extensions [l_9_ = 214 mm]) and two folded‐end coaxial‐end setups (0‐mm [l_1_ = 240 mm] and 20‐mm [l_1_ = 220 mm] gap), were compared in numerical simulations to identify the optimal head–neck dipole array design. For the optimal configuration, simple numerical 3D shimming was performed by applying an additional phase shift between the rows. Previously, it was demonstrated that this technique improved the RF field homogeneity and SAR performance [21, 28, 55].

### 2.2 Numerical Comparison With Loop Arrays and Other Dipole Arrays

For comparison, we also simulated two configurations of head and neck arrays based on loop elements (Duke voxel model only) and one dual‐row head‐only folded‐end dipole array constructed in our lab [55] (Duke voxel model and head and shoulder [HS] phantom [ε = 58.3, *σ* = 0.64 S/m]). The 16‐channel loop array was based on the ToRo 16Tx32Rx array [66] developed in our lab but extended in the longitudinal direction towards the shoulders to provide coverage of the brain and C‐spine as done previously to design a 7‐T head–neck array [43]. The 8‐channel loop array was based on a 7 T TxRx loop array [41], adapted to 9.4 T by adjusting the number and value of capacitors. Array sizes, number of distributed capacitors, and decoupling methods for these two arrays are presented in Figure S1. As with the dipole arrays, <B_1_^+^_ROI_>, COV in ROI, pSAR_10g_, and SAR efficiency in the ROI were calculated. Additionally, the same metrics were calculated separately for the brain and C‐spine regions.

### 2.3 Array Construction

**FIGURE 3:**
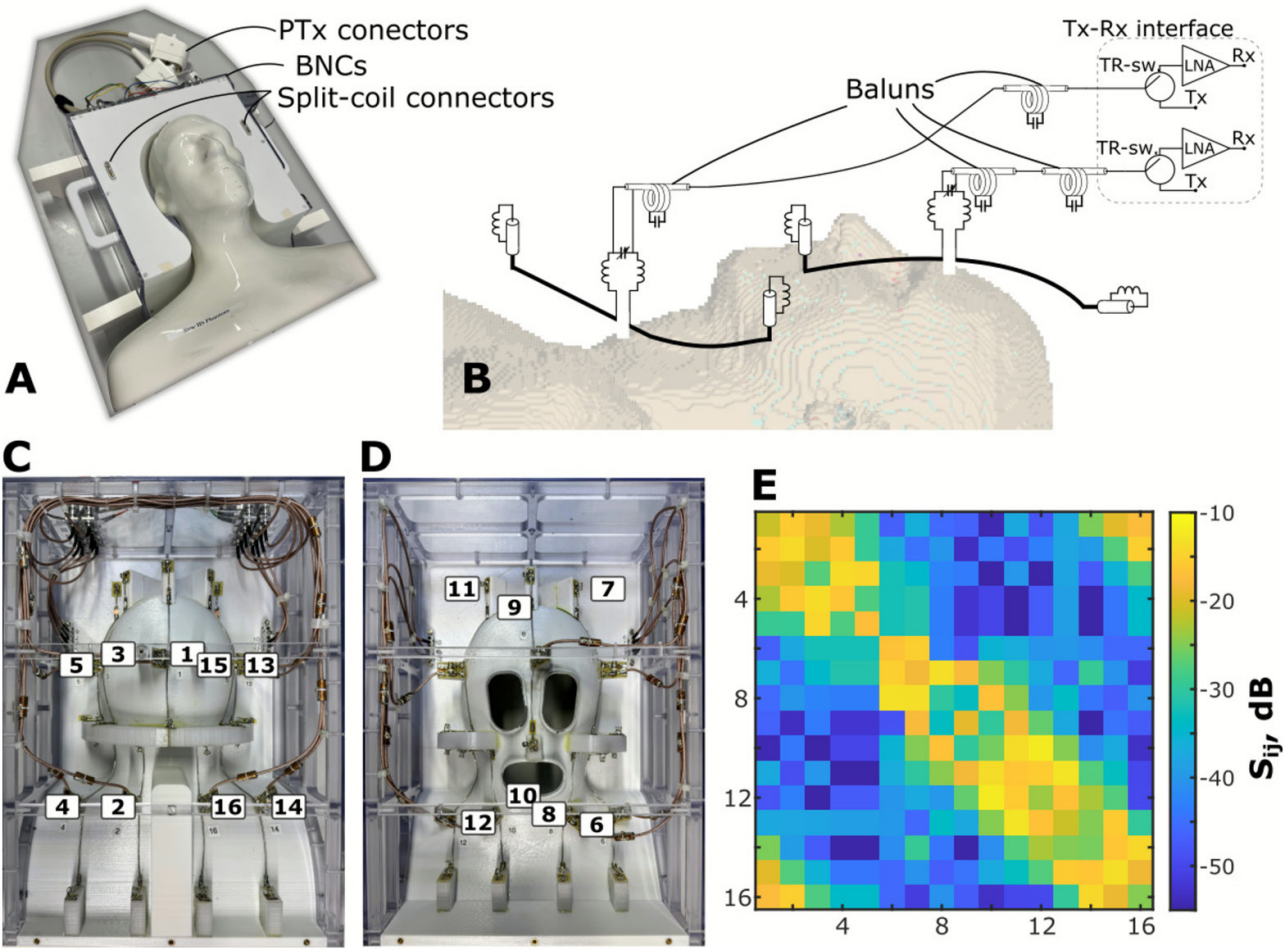
(A) Isometric photo of the constructed 16‐channel coaxial‐end array (upper part of the array is removed). (B) A simplified electrical circuit of a pair of coaxial‐end dipoles as used in the experiments. (C) Photo of the bottom part of the constructed array. (D) Photo of the top part of the constructed array. The displayed numbers enumerate the individual dipole elements. (E) Measured S‐matrix at 400 MHz of the array loaded with a healthy volunteer (Subject 1).

After finishing the numerical simulation and defining the optimal array geometry (folded‐end coaxial‐end, 10 mm extension, 20 mm gap between rows), the proposed array was constructed and evaluated on the bench. The array holder was 3D‐printed from polycarbonate using FDM technology (innovatiQ, Germany) and attached to the coil housing frame (Figure 3A,C,D) milled from polycarbonate plates. Afterwards, five subjects spent 1 h each inside the housing to ensure comfort and sufficient heat dissipation. Sixteen BNC flange‐mount connectors (Huber + Suhner, Switzerland) were placed on the bottom front flange of the coil to connect it with the home‐built TxRx 16‐channel MR‐scanner interface consisting of 16 high‐power TR‐switches [18] and 16 OEM low‐noise amplifiers (WanTcomm, USA). The bottom and top parts of the array were electrically connected using Split‐Coil (ODU, Germany) coaxial connectors. To connect the BNCs and array element, low‐loss coaxial cable (K_02252_D‐60, Huber+Suhner, Switzerland) was used. All cable lengths were adjusted to have the same (±1° phase variation) phase shift. Two floating ground cable traps [67] per cable prevented propagation of the common mode along the cable. Matching circuits were placed on small‐footprint PCBs made from FR‐4 material. The L‐matching network consisting of two series of self‐wound inductors (1.2‐mm wire diameter, Q ≈ 120) and a parallel high‐power variable capacitor (52H02, 1–19 pF, Johanson, USA) was used (Figure 3B). Supports for the dipole ends and matching circuits were 3D‐printed (Ultimaker, Netherlands) from transparent polycarbonate material (ε = 2.9, tgδ = 0.011). All coil elements were tuned and matched on a homogenous HS‐phantom mimicking human tissue properties at 400 MHz (ε = 58.6, *σ* = 0.64 S/m) [68]. To prevent the coil housing from movement during measurements, additional extensions of the coil housing (Figure 3A) in the form of the patient bed were printed from the polycarbonate material. The 3D mechanical design of the array assembly was uploaded to a repository [69] and is available for download.

### 2.4 MRI Experiments

For real‐time SAR management and monitoring, a set of virtual observation points (VOP) matrices (N_VOP_ = 200, safety factor 2.2) [70, 71] was calculated based on the array numerical simulations. An experimental study was performed on a head and shoulder phantom and three healthy volunteers with different head sizes (two males [58‐ and 57‐cm circumference] and one female [54‐cm circumference]). The local ethics committee approved the in vivo study, and informed consent was obtained from each subject prior to examination. Before the experiment, matching of the array was performed on the male volunteer with a relatively large head (58‐cm circumference) and tested on all other volunteers before each MR experiment without any further adjustments of tuning and matching. To determine the S‐matrix, a two‐port VNA Keysight ENA 5063A (Santa Rosa, California, USA) was used. While measuring S‐parameters of two elements, all other elements were loaded to 50‐Ohm loads.

All data were acquired using a Siemens MAGNETOM Plus 9.4 T (Siemens Healthineers, Germany) whole‐body scanner (16 Tx, 32 Rx). B_1_^+^ maps in the phantom were acquired using a 3D AFI sequence (TR = 20/100 ms, TE = 2 ms, excitation FA = 40°, matrix size 128 [HF] × 96 [AP] × 128 [LR], FOV 380 mm × 285 mm × 380 mm, bandwidth = 700 Hz/pixel) [72] and a 3D satTFL sequence (TR = 1/7/20262.5 ms, TE = 0.73 ms, excitation FA = 2°, saturation FA = 50°, GRAPPA 2 × 2, matrix size 64 [HF] × 48 [AP] × 64 [LR], FOV 380 mm × 285 mm × 380 mm, bandwidth = 700 Hz/pixel) [73]. In vivo T_1_‐weighted images were acquired using a 1‐mm‐isotropic MPRAGE sequence [62] (TR/TI = 3.36/1.34 s, US = 2 × 2, GRAPPA [74] reconstruction, nominal matrix size 364 [HF] × 242 [AP] × 192 [LR], adiabatic inversion pulse [HS4], excitation FA = 9°, bandwidth = 312 Hz/pixel). In vivo B_1_^+^ mapping was performed using the vendor‐provided 2D presaturated TurboFLASH sequence [75] (“tfl_b1map”, TR/TE = 10 s/2.1 ms, US = 2, GRAPPA reconstruction, FOV = 380 mm × 262 mm, 28 sagittal slices of 3‐mm thickness, base matrix size 64, in‐plane resolution 6 mm × 6 mm, twofold zero‐filling interpolation, saturation FA = 90°, excitation FA = 8°, bandwidth = 485 Hz/pixel). Based on the known nominal reference voltage, the acquired flip angle maps were converted into maps of B_1_^+^ per input power. In vivo measurement of SNR was performed following the method from [76]. For this, a non‐accelerated 3D gradient and RF‐spoiled GRE (TR = 6 ms, TE = 3 ms, excitation FA = 5°, matrix size 380 [HF] × 286 [AP] × 384 [LR], FOV 380 mm × 285 mm × 380 mm, bandwidth = 450 Hz/pixel) dataset was acquired as well as a noise‐only scan for which the reference voltage was set to zero but gradients were still played out. Then, calculation of SNR in absolute units was performed offline using custom‐written MATLAB scripts. Since reconstructions for SNR calculation were performed offline, the obtained SNR distributions are not distortion‐corrected. B_1_^+^ distributions on the phantom, in vivo GRE images and SNR were also measured using a dual‐row head‐only dipole array [55] for comparison with proposed head–neck array. For all other scans, the vendor‐provided online 3D geometric distortion correction was applied to compensate for gradient nonlinearity over the large field of view.

Brain masks were obtained from the FSL Brain Extraction Tool [77], and spinal cord masks were created using the Spinal Cord Toolbox [78, 79], both of which were applied to the MPRAGE images. For display purposes, the spinal cord masks were dilated by a sphere of 6‐mm radius and merged with the brain masks to obtain a joint mask of the relevant anatomical regions.

## 3 Results

### 3.1 Numerical Simulations

#### 3.1.1 Straight Dipole Arrays

**FIGURE 4:**
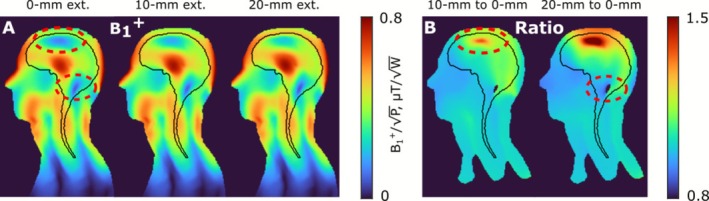
(A) Numerically calculated B_1_^+^ (normalized to 1 W of stimulated power) distributions created by the straight coaxial‐end (l_1_ = 194 mm) 16‐channel array in the central sagittal slice of the Duke voxel model. The ROI is marked with a black line. (B) The ratio of B_1_^+^ of the 10‐mm (l_1_ = 204 mm) and 20‐mm (l_1_ = 214 mm) extended array to B_1_^+^ of the non‐extended (0‐mm, l_1_ = 194 mm) array fields in a central sagittal slice of the Duke voxel model.

Figure 4A shows numerically calculated B_1_^+^ maps for the Duke voxel model and straight array configurations with 0‐mm (initial configuration to cover 365‐mm region, l_1_ = 194 mm), 10‐mm (l_1_ = 204 mm), and 20‐mm (l_1_ = 214 mm) extensions of the dipoles above the head. As seen from the field distributions, the initial configuration of the array provides excitation over the whole ROI. However, two dropouts could be observed: one at the top of the brain (the superior location) and the other one at the cerebellum region. Extending the length of dipoles above the head by 10 mm improves B_1_^+^ in both problematic regions. Extending the dipoles further to 20 mm increases the field at the top of the head but reduces the field at the cerebellum region. To compare the 10‐mm (l_1_ = 204 mm) and 20‐mm extended (l_1_ = 214 mm) array configurations to the non‐extended (0‐mm) one, we calculated the respective ratios of the B_1_^+^ distributions (Figure 4B). Table 1 shows quantitative data including B_1_^+^ averaged over the ROI (indicated by black solid line in Figure 4), B_1_^+^ COV over the ROI, pSAR_10g_, and SAR efficiency. According to the results presented in the table, the 20‐mm extended array performs better than the other array configurations. For example, pSAR_10g_ for the 20‐mm array is 26% lower than for the 0‐mm array and 19% lower than for the 10‐mm array; SAR efficiency is 18% higher than for 0‐mm and 10% higher than for 10 mm. The COV for the 20‐mm extended array was also lower than for the 0‐mm (12%) and 10‐mm array (2%). However, since the cerebellum region is critical for combined brain and C‐spine imaging, we chose the 10‐mm extended array for further investigation. All array configurations demonstrated good decoupling performance: Worst decoupling values were −9.7 ± 0.2 dB for all arrays.

**TABLE 1:** Numerically calculated Tx efficiency (mean B_1_^+^ over ROI normalized to the square root of stimulated power), pSAR_10g_, SAR efficiency (mean B_1_^+^ over ROI normalized to the square root of pSAR_10g_), and COV (standard deviation of B_1_^+^ normalized to mean B_1_^+^ over ROI) for the Duke voxel model for different array configurations.

Array	<B_1_^+^>, μT/√W	<B_1_^+^>, rel. to opt.	pSAR_10g_, W/kg	pSAR_10g_, rel. to optimal	SAR_eff_, μT/√W/kg	SAR_eff_, rel.to optimal	COV	1/COV, rel. to optimal
Straight (l_1_ = 194 mm) (0 deg b/w rows)	0.365	0.95	0.571	1.64	0.483	0.74	0.335	0.93
Straight, 10 mm ext. (l_1_ = 204 mm) (0 deg b/w rows)	0.378	0.98	0.539	1.19	0.515	0.79	0.324	0.97
Straight, 20 mm ext. (l_1_ = 214 mm) (0 deg b/w rows)	0.382	0.99	0.452	1.30	0.571	0.88	0.318	0.99
Folded‐end, 0 mm gap (l_1_ = 240 mm) (0 deg. b/w rows)	0.344	0.90	0.369	1.06	0.566	0.87	0.289	1.09
Folded‐end, 20‐mm gap (optimal) (l_1_ = 220 mm) (0 deg b/w rows)	0.384	1	0.348	1	0.651	1	0.314	1
Folded‐end, 20‐mm gap (−60 deg b/w rows)	0.267	0.69	0.477	1.37	0.389	0.60	0.351	0.89
Folded‐end, 20‐mm gap (−30 deg b/w rows)	0.322	0.84	0.423	1.27	0.496	0.76	0.316	0.99
Folded‐end, 20‐mm gap (0 deg b/w rows)	0.384	1	0.348	1	0.651	1	0.314	1
Folded‐end, 20‐mm gap (30 deg b/w rows)	0.436	1.14	0.354	1.02	0.733	1.12	0.323	0.97
Folded‐end, 20‐mm gap (60 deg b/w rows)	0.469	1.22	0.441	1.26	0.725	1.11	0.351	0.89

In the next step, we simulated the 10‐mm extended array using the Ella voxel model. For this simulation, no additional adjustment of matching circuits was performed. Numerically simulated reflection coefficients (S_ii_) measured at the array inputs demonstrated strong shifts of the elements' resonant frequencies (i.e., frequencies where the minimum input reflection is observed). Bar plots with frequency shifts from the Duke to the Ella voxel model are presented in Figure S2A. The value of the input matching (S_ii_) at 400 MHz for Ella is shown in Figure S2B. These two plots show that the frequency shifts can be more than 25 MHz. Such a strong shift of the resonant frequency leads to an input matching worse than −3 dB and strong reflection. Since, during in vivo studies, it is highly desirable to avoid adjusting coil tuning and matching for every volunteer, such an array configuration is unsuitable for our application scenario of combined brain and C‐spine imaging. The 10‐mm extended straight coaxial‐end array configuration was modified to a folded‐end coaxial‐end array to overcome these strong load sensitivity issues.

#### 3.1.2 Folded‐End Dipole Arrays

**FIGURE 5:**
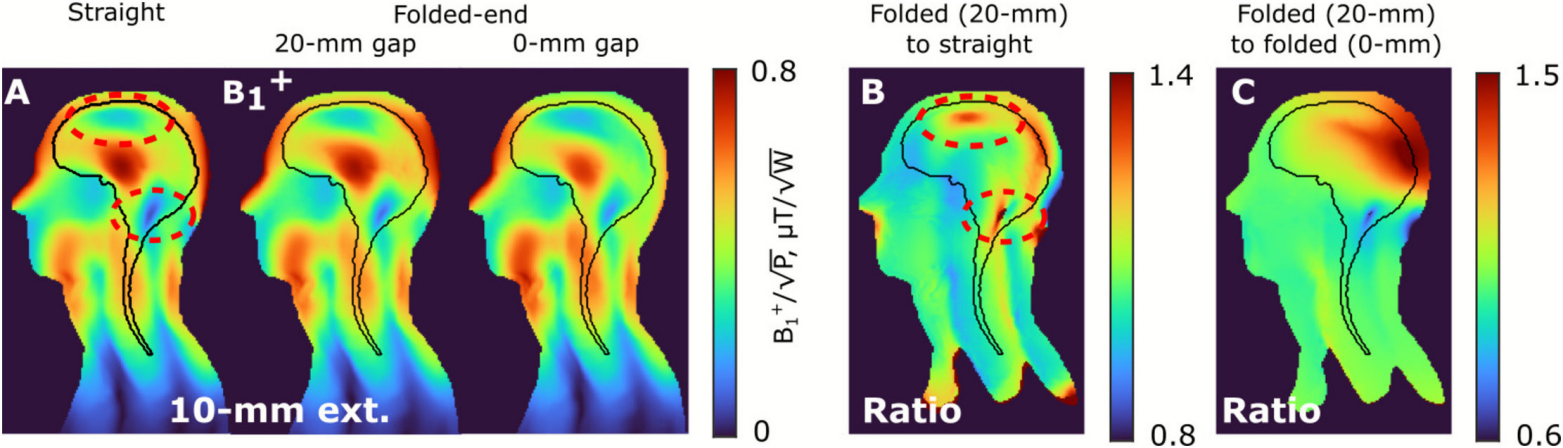
(A) Numerically calculated B_1_^+^ (normalized to 1 W of stimulated power) distributions created by the folded‐end coaxial‐end and two configurations of folded‐end (0‐mm [l_1_ = 240 mm] and 20‐mm [l_1_ = 220 mm] gap between rows) 16‐channel arrays in the central sagittal slice of the Duke voxel model. The ROI is marked with a black line. (B) The ratio of B_1_^+^ of the straight dipole array to B_1_^+^ of the folded‐end array with a 20‐mm gap between the rows and the ratio of B_1_^+^ of the folded‐end array with a 20‐mm gap to an array of folded‐end dipoles with a 0‐mm gap in the central sagittal slice of the Duke voxel model.

Numerically simulated B_1_^+^ maps in the central sagittal slice of the Duke voxel model for the folded‐end coaxial‐end dipole array in two configurations (0‐mm gap [l_1_ = 240 mm] and 20‐mm gap [l_1_ = 220 mm]) are presented in Figure 5A. A B_1_^+^ map in the same slice for the straight 10‐mm coaxial‐end dipole array configuration is presented alongside for comparison. The presented field distributions show that the array with the 0‐mm gap between the rows has suboptimal performance. A significant drop in the B_1_^+^ field compared to the straight and 20‐mm gap folded‐end coaxial‐end array in the parietal and occipital lobe regions can be noticed. For an additional illustration of the folded‐end coaxial‐end array performance, we calculated ratios of the B_1_^+^ map of the folded‐end coaxial‐end array with the 20‐mm gap to B_1_^+^ maps of the straight (l_9_ = 194 mm) and 0‐mm gap folded‐end coaxial‐end (l_9_ = 240 mm) arrays (Figure 5B). These ratios confirmed the results of Figure 5A. This effect can be observed because the dipoles placed in the upper row of the 0‐mm gap array almost reach their self‐resonant frequency. As demonstrated in [56], such a dipole array would generate a less homogeneous B_1_^+^ field in the sample. In addition, the worst measured coupling for the 0‐mm (l_1_ = 220 mm) and 20‐mm (l_1_ = 220 mm) gap folded‐end coaxial‐end arrays loaded by the Duke voxel model was −8.56 and −8.99 dB, respectively, which are slightly worse than that obtained for the straight array.

After choosing the folded‐end coaxial‐end array design, i.e., the array with a 20‐mm gap between the rows, we evaluated numerically the resonant frequency shift and matching when replacing the Duke by the Ella voxel model. Bar plots with the corresponding results are presented in Figure S2A,B. As seen in Figure S2A, phase shifts obtained for the folded‐end coaxial‐end array are significantly lower than those for the straight one. Also, the worst measured matching for Ella was better than −9.5 dB. Based on this result, we chose the 20‐mm gap/10‐mm extended folded‐end coaxial‐end array configuration for future investigations.

#### 3.1.3 Optimization of Inter‐Row Phase Shift

**FIGURE 6:**
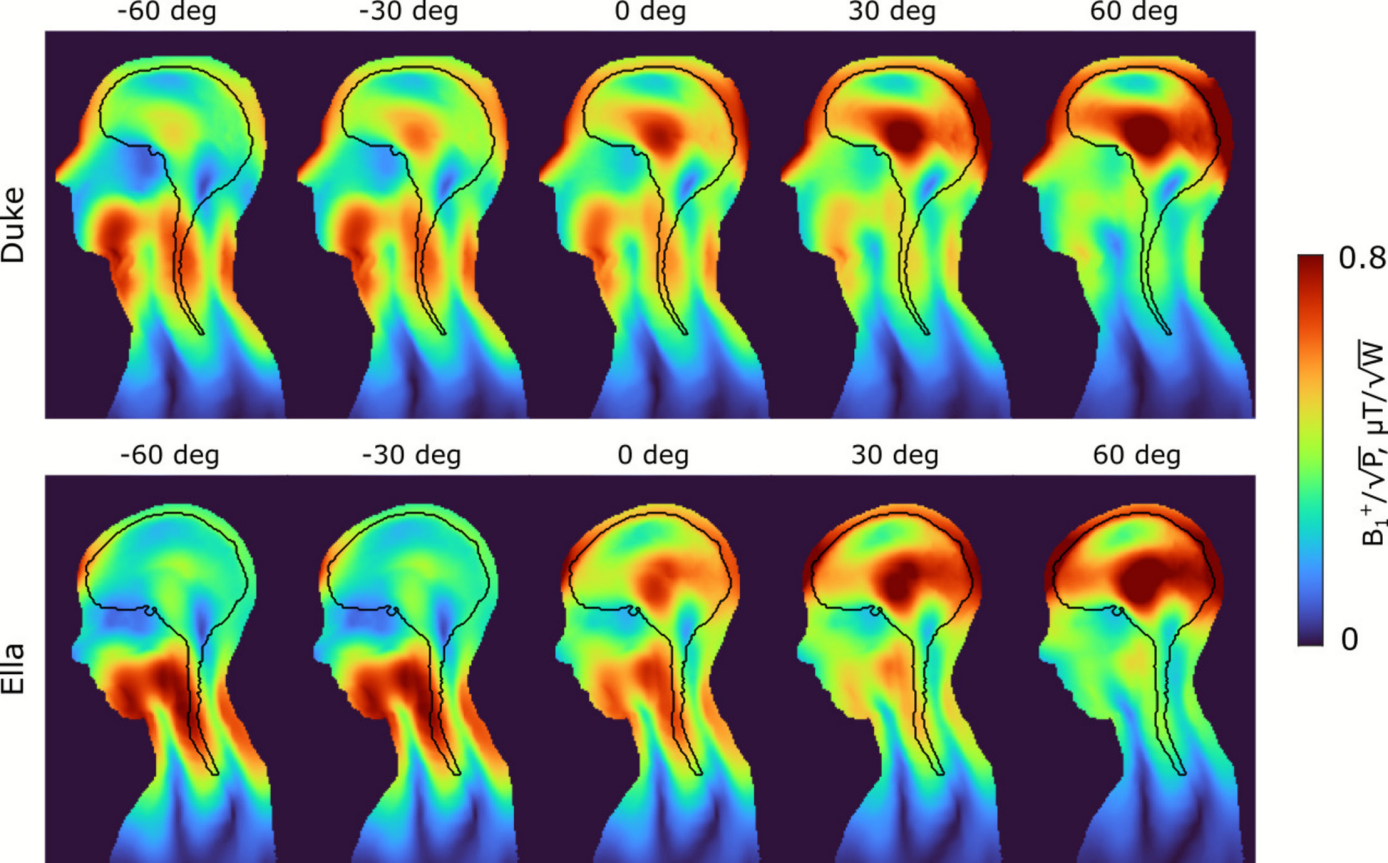
(A) Numerically calculated B_1_^+^ (normalized to 1 W of stimulated power) distributions created by the folded‐end coaxial‐end (10‐mm extended, 20‐mm gap between rows, l_1_ = 220 mm) 16‐channel array in the central sagittal slice of the Duke and Ella voxel models for different phase shifts between the rows.

**FIGURE 7:**
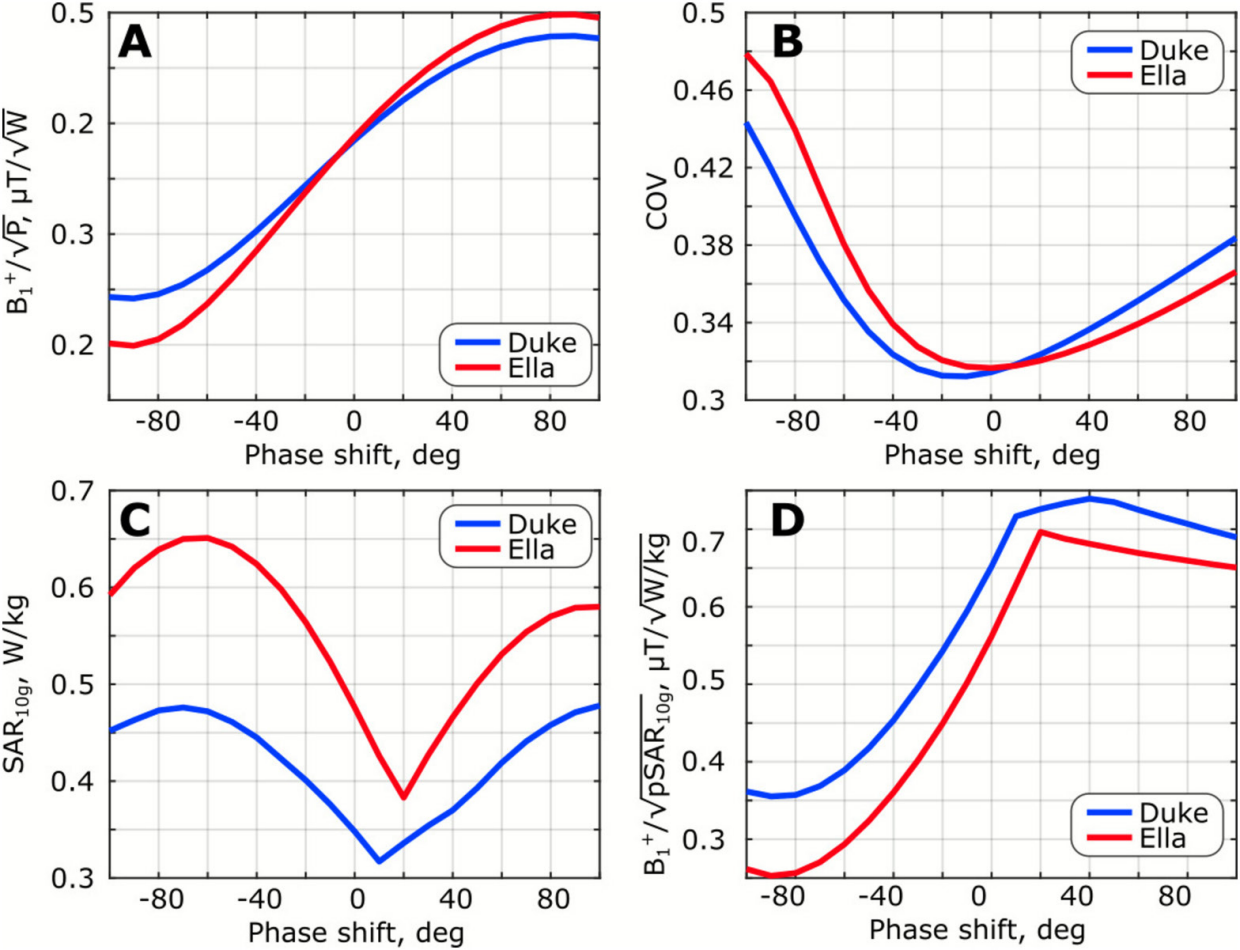
Numerically calculated Tx efficiency (mean B_1_^+^ over the ROI normalized to the square root of stimulated power) (A), COV (standard deviation B_1_^+^ normalized to mean B_1_^+^ over the ROI) (B), pSAR_10g_ (C), and SAR efficiency (mean B_1_^+^ over the ROI normalized to the square root of pSAR_10g_) (C) for the Duke and Ella voxel models. All simulations were performed for a folded‐end coaxial‐end 16‐channel array (10‐mm extended, 20‐mm gap between rows, l_1_ = 220 mm).

B_1_^+^ distributions created by the 20‐mm (l_1_ = 220 mm) gap folded‐end coaxial‐end array in the central sagittal slice of the Duke and Ella voxel models for different phase shifts between the rows are presented in Figure 6. Tx efficiency and COV calculated over the ROI (marked with a black contour in Figure 6) are presented in Figure 7A,B, respectively. The values of pSAR_10g_ and SAR efficiency are presented in Figure 7C,D. These data are also presented in Table 1 for the Duke voxel model. As seen in Figure 7 and Table 1, the Tx efficiency increases with an increase in the phase shift between the rows. However, COV minima appear very close to the 0° phase shift. pSAR_10g_ minima appear for phase shifts of 10° and 20° for the Duke and Ella voxel models, respectively. The SAR‐efficiency trend, in general, follows the Tx efficiency. Since our primary goal was the best excitation homogeneity, we used a case with no phase shift in further in vivo experiments.

#### 3.1.4 Numerical Comparison Between Dipole and Loop Arrays

**FIGURE 8:**
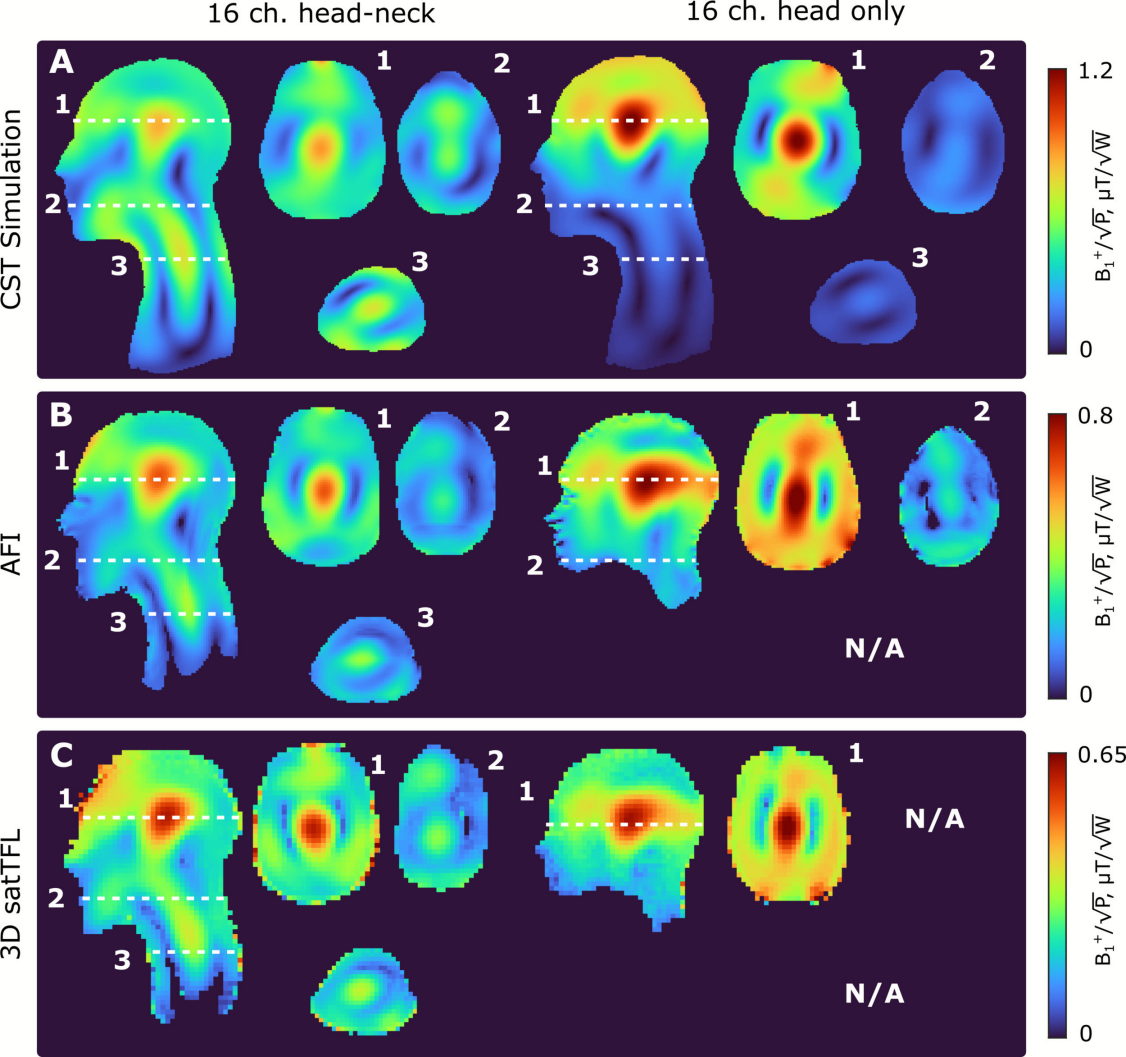
(A) Numerically calculated and measured B_1_^+^ maps using AFI (B) and 3D satTFL (C) sequences (normalized to 1 W of stimulated power) distributions created by the folded‐end coaxial‐end 16‐channel head–neck array and 16‐channel folded‐end head‐only dipole array using head and shoulder phantom.

**FIGURE 9:**
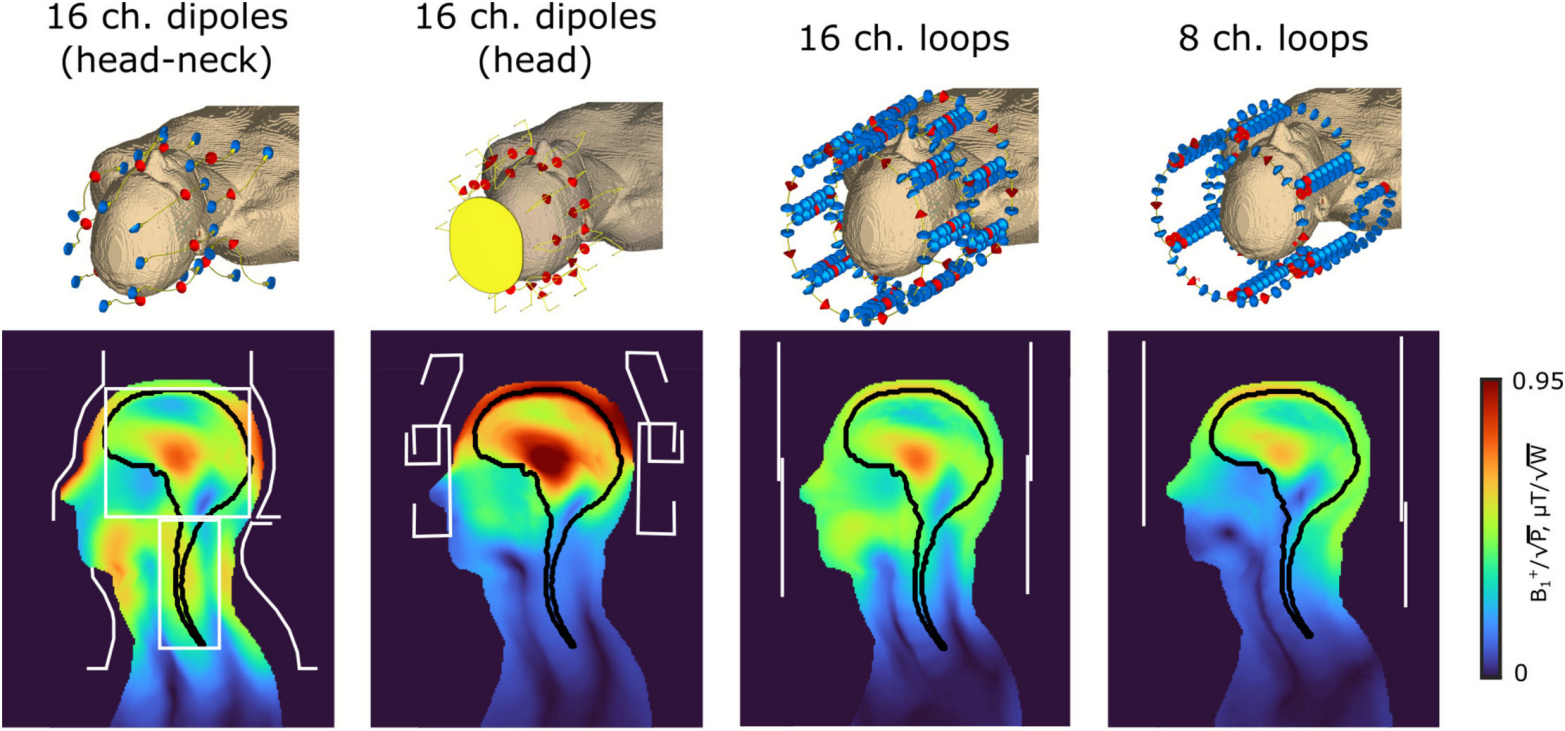
Isometric view of numerical models of 16‐channel head–neck and head‐only dipole array and 16‐ and 8‐channel loop arrays in CST Studio 2021. Numerically calculated B_1_^+^ (normalized to 1 W of stimulated power) distributions created by 16‐channel dipole and 16‐ and 8‐channel loop arrays in the central sagittal slice of the Duke voxel model. The full ROI is marked with a solid black line, while the brain and C‐spine regions used for separate <B_1_^+^> and COV calculations are marked with a black dashed line.

A comparison of the transmit efficiency of the developed 16‐channel folded‐end coaxial‐end head–neck array and a 16‐channel head‐only folded‐end dipole array in the HS‐phantom is presented in Figure 8A. In addition, a comparison of B_1_^+^ distributions, created in the central sagittal slice of the Duke voxel model by the folded‐end coaxial‐end 16‐channel dipole array, 16‐channel folded‐end dipole array, 16‐channel loop array, and 8‐channel loop array, is presented in Figure 9. <B_1_^+^_ROI_> and COV calculated over the full ROI (brain and C‐spine), over the brain only, and over the C‐spine only, as well as pSAR_10g_ and SAR efficiency (over different ROIs) are presented in Table 2. As seen in Figure 9 and Table 2, the proposed dipole array outperforms the loop arrays with cylindrical geometry in terms of Tx efficiency and field homogeneity. However, pSAR_10g_ is lower for the loop array since the dipoles are located much closer to the tissue. This results in a slightly higher SAR efficiency for the loop arrays when calculated over the full ROI and brain only. However, the dipole array outperforms substantially both loop arrays in terms of Tx efficiency and SAR efficiency calculated over the C‐spine region.

**TABLE 2:** Numerically calculated Tx efficiency (mean B_1_^+^ over full ROI/brain/C‐spine, normalized to the square root of stimulated power), pSAR_10g_, SAR efficiency (mean B_1_^+^ over ROI/brain/C‐spine normalized to the square root of pSAR_10g_) and COV (standard deviation of B_1_^+^ normalized to mean B_1_^+^ over ROI/brain/C‐spine) for the Duke voxel model for different dipole and loop arrays.

Array and region	<B_1_^+^>, μT/√W	<B_1_^+^>, rel. to proposed	pSAR_10g_, W/kg	pSAR_10g_, rel. to proposed	SAR_eff_, μT/√W/kg	SAR_eff_, rel. to proposed	COV	1/COV, rel. to proposed
16 dipoles (proposed)—brain	0.383	1	0.348	1	0.650	1	0.315	1
16 dipoles (head array)—brain	0.541	1.41	0.484	1.39	0.778	1.20	0.31	1.02
16 loops—brain	0.353	0.92	0.254	0.73	0.701	1.08	0.335	0.94
8 loops—brain	0.329	0.86	0.216	0.62	0.709	1.09	0.352	0.89
16 dipoles (proposed) C‐spine	0.451	1	0.348	1	0.766	1	0.256	1
16 dipoles (head array) C‐spine	0.172	0.38	0.484	1.39	0.247	0.32	0.268	0.95
16 loops—C‐spine	0.306	0.68	0.254	0.73	0.609	0.80	0.272	0.94
8 loops—C‐spine	0.213	0.472	0.216	0.62	0.459	0.60	0.319	0.80

### 3.2 Experimental Study

**FIGURE 10:**
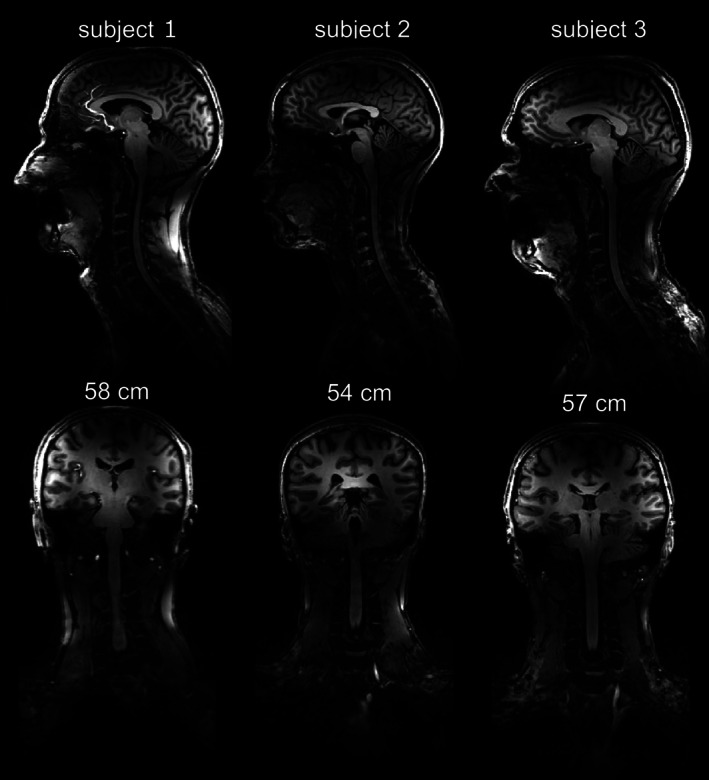
MPRAGE images of three healthy volunteers in central coronal and sagittal slices with gradient nonlinearity correction applied. Numbers indicate the respective subject's head circumference.

An example of the full measured S‐matrix of the developed array loaded with a 31‐year‐old healthy male volunteer (Figure 10, Subject 1) is presented in Figure 3E. The full S‐matrix for a 28‐year‐old healthy volunteer (Figure 10, Subject 2) and a 31‐year‐old healthy volunteer with head size larger than Subject 1 (Subject 4) is depicted in Figure S3A. Measured S_ii_ values (input matching) for three volunteers are shown in Figure S3B. All array elements were matched better than −11 dB. No further adjustments of tuning and matching were performed after initial tuning and matching on Subject 1. The worst coupling measured on Subject 1 was −11.7 dB between adjacent elements in the bottom row.

**FIGURE 11:**
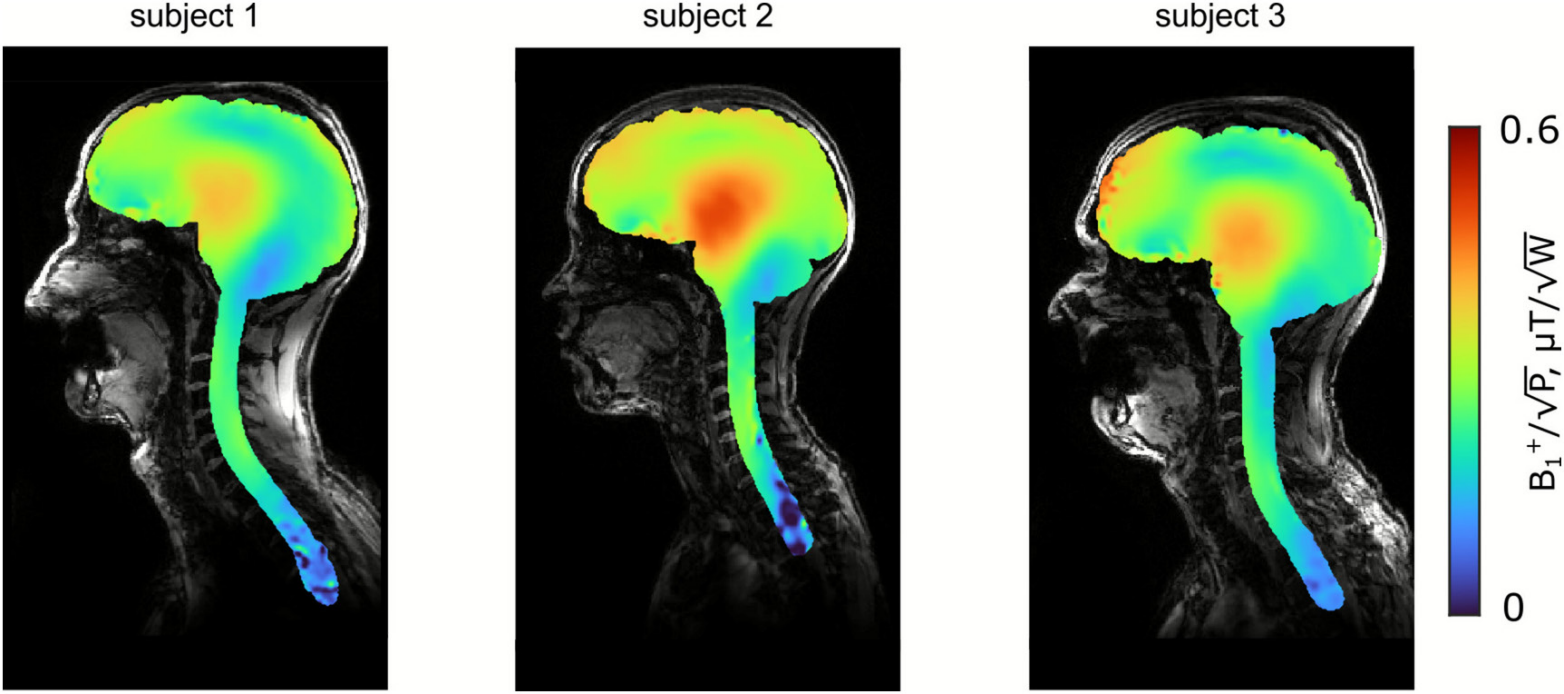
B_1_^+^ maps acquired using the vendor‐provided presaturated TurboFLASH sequence for the three healthy volunteers displayed in a central sagittal slice overlaid on the MPRAGE images.

Figure 8B,C shows measured B_1_^+^ distributions in the central sagittal and three transversal planes (roughly corresponding to position hypothalamus, C1, and C5 in healthy human) for head–neck and head‐only dipole arrays using AFI and satTFL sequences. From this plot, we can clearly see that the proposed array can provide large FOV excitation, predicted by numerical simulations. Figure 10 shows anatomical MPRAGE images of three subjects, demonstrating coverage of the entire cerebrum, cerebellum, and C‐spine down to C7 and beyond. Figure 11 displays the corresponding CP mode B_1_^+^ maps as overlays on the MPRAGE images. B_1_^+^ maps without image overlaying are presented in Figure S4. The peak B_1_^+^ efficiencies at the center of the cerebrum were 0.40, 0.50, and 0.43 μT/√W for Subjects 1, 2, and 3, respectively, showing an inverse dependency on the head size. Along the C‐spine (between C1 and C7), the proposed array provides B_1_^+^ in the range from 0.15 to 0.2 μT/√W, showing no significant dropouts.

**FIGURE 12:**
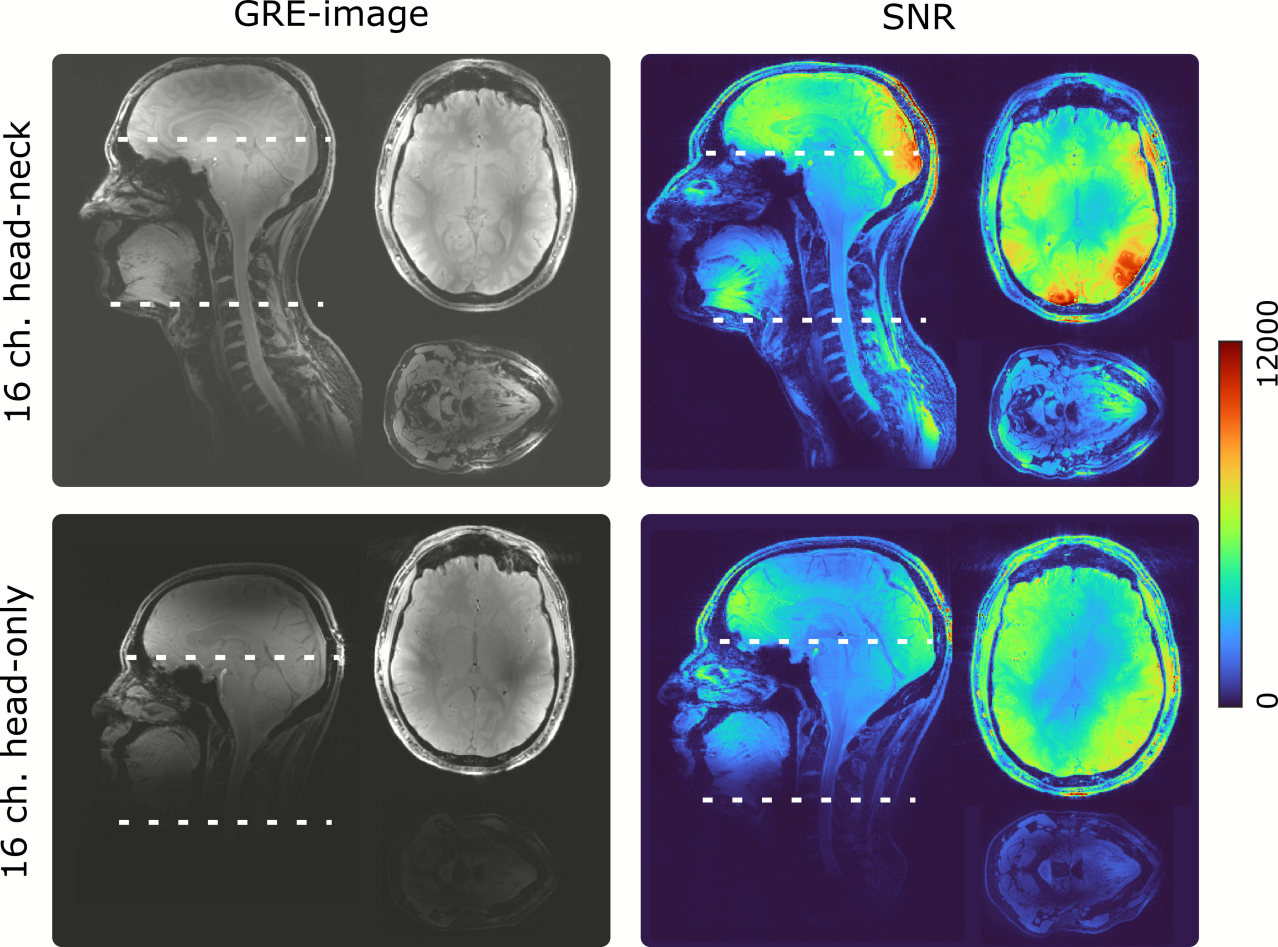
3D GRE images of a healthy volunteer (Subject 4) and corresponding measured SNR distributions obtained using the proposed 16‐channel head–neck array and using the 16‐channel head‐only dipole array.

Measured GRE images as well as SNR for head–neck and head‐only 16‐channel transceiver arrays are presented in Figure 12. Overall, SNR of the proposed head–neck array is higher than SNR of the head‐only array not only in the C‐spine but also in the brain region.

## 4 Discussion

In this work, a 16‐channel dual‐row TxRx folded‐end coaxial‐end dipole array for combined brain and C‐spine MRI at 9.4 T was designed, constructed, and evaluated numerically and experimentally. Since a homogeneous Tx coverage over a very large area (more than 35 cm longitudinally) is a crucial but very challenging task, the primary goal of this study was to develop a prototype of a TxRx head–neck array and evaluate its Tx performance. With the proposed array design, more than 365 mm of longitudinal coverage is achieved, allowing simultaneous anatomical imaging of the brain (including motor cortex, the cerebellum, and brainstem) and C‐spine with the largest field of view yet realized on a 9.4 T human scanner. By optimizing the length and shape of the dipoles, we maximized B_1_^+^ homogeneity and minimized the sensitivity (the frequency shift) of the array to loading. In contrast to recently published studies on UHF with similar coil designs [41, 42], where dual‐row transmit loop arrays were used, we used a relatively tight‐fit dipole array.

To compare the performance of the proposed dipole array coil with that of loop arrays, we adapted the design of the previously reported 7 T head–neck array [41] for 9.4 T. However, since this array has only eight channels, a comparison with 16‐channel dipole arrays is not entirely fair. Therefore, we also designed a 16‐channel loop array with a similar longitudinal length as the 8‐loop array. Our numerical simulations show that cylindrical loop‐based arrays cannot provide good excitation over the C‐spine region (Table 2). Tx performance of the loop array over C‐spine could be potentially improved by using a 2 × 8 Tx array with the ergonomically shaped holder similar to one presented in work [42]. However, such an array would require 16 long (~200‐mm) loops. According to our simulations (first row of the 8‐channel loop array; Figure S1B), such a long loop requires a significant number of capacitors (~30) to be distributed along the conductors bringing the total number of high‐voltage capacitors to a very high value of ~500. Still, each capacitor measures only 4.5 pF, which is comparable to the parasitic capacitance of the loop itself. Using dipoles eliminates the necessity of numerous distributed capacitors and significantly simplifies the coil design, making it more robust and reliable.

In comparison to the previously designed dual‐row folded‐end dipole array [55], the developed array does not require additional decoupling circuits. Lower intrinsic coupling between the adjacent dipoles is associated with the difference in the dipole designs, i.e., replacing the folded‐end wires, which produce a strong electrical field and couple to each other capacitively [59], by short coaxials at the dipole's ends. Performance of the proposed folded‐end coaxial‐end array was also compared to a folded‐end 2 × 8 head‐only array both numerically and experimentally in a homogenous phantom and in vivo. In general, good agreements between simulated and measured B_1_^+^ distributions were observed (Figure 8). Tx efficiency of the head‐only array was higher in the brain region with a fast drop of B_1_^+^ towards the C‐spine region. Experimental in vivo scans using MPRAGE and GRE sequences confirm the results of the numerical simulations on three healthy volunteers. C‐spine coverage is sufficient to enable anatomical imaging down to C7 and beyond for all measured volunteers (Figure 10). Measured SNR of the head–neck array was higher in the brain region than that of the head‐only array (Figure 12), due to tighter fit and, therefore, better loading of the array elements. In addition, the thoroughly designed ergonomically shaped array housing provides increased patient comfort.

Initially, we designed a straight coaxial‐end array that was demonstrated previously to have lower sensitivity to loading than the common straight dipoles [56]. However, because of the tight‐fit array configuration, we still found a strong frequency shift occurring with the load variation (Figure S2). Folding the coaxial ends away from the subject helped to minimize this effect (Figure S2). Also, similar to arrays previously designed in our laboratory [51, 53, 55, 59], extending the dipoles above the top of the head (the superior location) improves RF field homogeneity. However, considering the dual‐row arrangement of dipole elements, we found that an extension of more than 20 mm leads to a decrease in the field in the cerebellum region. Therefore, we limited the extension to 10 mm, which increases the field in the superior brain region without compromising the field in the cerebellum.

In the present work, RF excitation was performed in the CP mode with an optimizable phase shift between the two rows. According to numerical simulations, the best homogeneity (i.e., the lowest COV) was achieved for an additional phase shift close to 0°. As reported previously, static RF shimming for large 3D volumes such as the combined brain and cervical spine region has only limited potential to improve homogeneity compared to the CP mode [21]. Thus, in the future, we plan to investigate more advanced pTx methods such as spokes [80], kT‐points [81], and universal pulses [82]. For 2D imaging, static slice‐wise RF shimming has been successfully demonstrated targeting only the C‐spine at 7 T [35, 83, 84]. Nevertheless, it was recently found that the CP mode performed surprisingly well for C‐spine imaging across multiple subjects and sites, even when compared to individually tailored RF shim solutions [83]. A suggested reason for this observation is the dependence on reliable single‐channel B_1_^+^ maps for optimization of RF shims, which are difficult to obtain due to strong B_0_ inhomogeneities, subject motion, and partial volume effects in the C‐spine region. Therefore, robust B_1_^+^ mapping and shimming in the spinal cord at UHF is an area of active research [85] that can be further explored in the context of arrays for combined brain and C‐spine imaging, such as proposed here.

The ability to image the entire brain and C‐spine simultaneously could be particularly promising for combined functional MRI studies of the cortex, brainstem, and spinal cord, e.g., in the context of functional connectivity [8], pain processing [86, 87], or motor sequence learning [88]. Due to the lack of suitable arrays at UHF, such studies have been mainly performed at 3 T so far. Thus, the presented array (in combination with additional receive‐only loops) design may contribute to the perspective of translating studies of the central nervous system to UHF and taking advantage of the associated benefits of higher SNR and CNR. The proposed transceiver array design could also be adapted to different field strengths, higher and lower than 9.4 T. In addition, the absence of an electrical connection and an RF shield allowed splitting the array housing into two halves, providing easy access and comfort for subjects. This is also beneficial for future fMRI applications by facilitating visual access and placement of eye‐tracking equipment. The open, splitable design furthermore simplifies the placement of additional equipment inside the array, e.g., EEG caps.

The present work focuses on optimizing the transmit performance over the brain and C‐spine using a 16‐element TxRx array. The use of only 16 elements during signal reception certainly does not provide optimal SNR and limits the capabilities of parallel imaging and therefore undersampling. To further improve SNR, in the next step, we plan to add multiple Rx‐only loops similar to what is suggested in [31]. A combined hybrid TxRx‐dipole/Rx‐loop array will also benefit from increased SNR near the head center due to the dipole's contribution to the signal reception [44, 45]. Beyond the influence of the array design, there is still considerable room for improvement in image quality by optimizing pulse sequences and especially B_0_‐shimming [89, 90, 91].

## 5 Conclusion

We designed, constructed, and evaluated the 16‐channel transceiver folded‐end coaxial‐end dipole array for combined brain and C‐spine imaging at 9.4 T numerically and experimentally. In the experimental study, we obtained the first (to the best of our knowledge) combined images of the whole brain and C‐spine down to the C7 region at 9.4 T. In the future, to increase the excitation homogeneity, we plan to apply pTx dynamic shimming methods. Also, to improve SNR, we plan to combine TxRx dipoles with multiple Rx‐only loops.

## Author Contributions

**G. A. Solomakha:** conceptualization, investigation, numerical simulations, prototype manufacturing, data acquisition, validation, writing. **F. Glang:** conceptualization, methodology, software, data acquisition, writing. **M. W. May:** resources, prototype manufacturing, writing – review and editing. **S. Mueller:** methodology, software, data acquisition, writing – review and editing. **J. Walzog:** resources, prototype manufacturing, writing – review and editing. **A. Aghaeifar:** data acquisition, writing. **D. Bosch:** methodology, data acquisition, writing. **J. Bause:** supervision, writing – review and editing. **O. Kraff:** supervision, writing – review and editing. **K. Scheffler:** funding acquisition, project administration, resources, supervision, writing – review and editing. **H. H. Quick:** funding acquisition, project administration, resources, supervision, writing – review and editing. **N. I. Avdievich:** conceptualization, investigation, prototype manufacturing, funding acquisition, project administration, resources, supervision, writing – review and editing.

## Supporting information


**Figure S1:** Design of the numerical model of the 16‐channel loop array (A) and 8‐channel loop array (B) used for comparison with the optimal configuration of the dipole array (folded‐end/10‐mm extended/20‐mm gap). Transformer decoupling was implemented for decoupling between the elements in one row, and overlapping decoupling was implemented for decoupling between the rows.
**Figure S2:** (A) Numerically calculated frequency shift of 20‐mm gap array element input reflection coefficients when replacing the Duke with the Ella voxel model for straight and folded‐end 16‐channel arrays. (B) Numerically calculated matching for straight and folded‐end 16‐channel arrays loaded to the Ella model, but initially tuned and matched on Duke.
**Figure S3:** (A) Measured full S‐matrix at 399.72‐MHz frequency for three healthy volunteers. (B) Bar plot of S_ii_ (i.e., matching) for different volunteers.
**Figure S4:** B_1_^+^ maps acquired using the vendor‐provided presaturated TurboFLASH sequence for the three healthy volunteers displayed in a central sagittal slice.

## Data Availability

The data that support the findings of this study are available from the corresponding author upon reasonable request.
