# Supplementary material for: Transceiver 16‐Channel Coaxial‐End Dipole Array for Combined Head and C‐Spine MRI at 9.4 T
Source: NMR Biomed. 2026 Jan 26;39(3):e70228. doi: 10.1002/nbm.70228 (PMC12835457; doi:10.1002/nbm.70228)

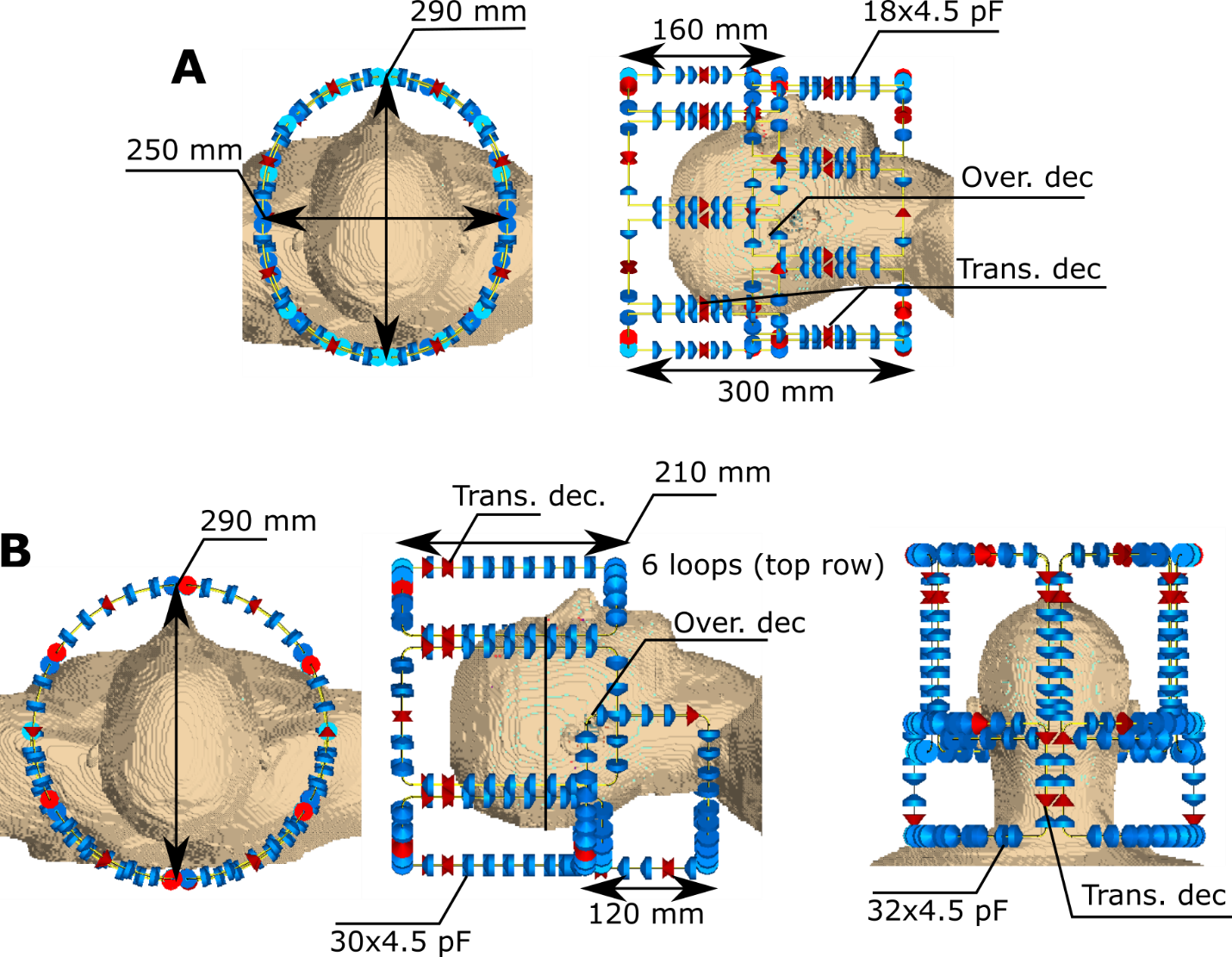


Figure S1. Design of the numerical model of the 16-channel loop array (A) and 8-channel loop array (B) used for comparison with the optimal configuration of the dipole array (folded-end/10-mm extended/20-mm gap). Transformer decoupling was implemented for decoupling between the elements in one row, and overlapping decoupling was implemented for decoupling between the rows.

Figure S3. Figure S3. (A) Measured full S-matrix at 399.72 MHz frequency for 3 healthy volunteers. (B) Bar plot of S_ii_ (i.e. matching) for different volunteers.


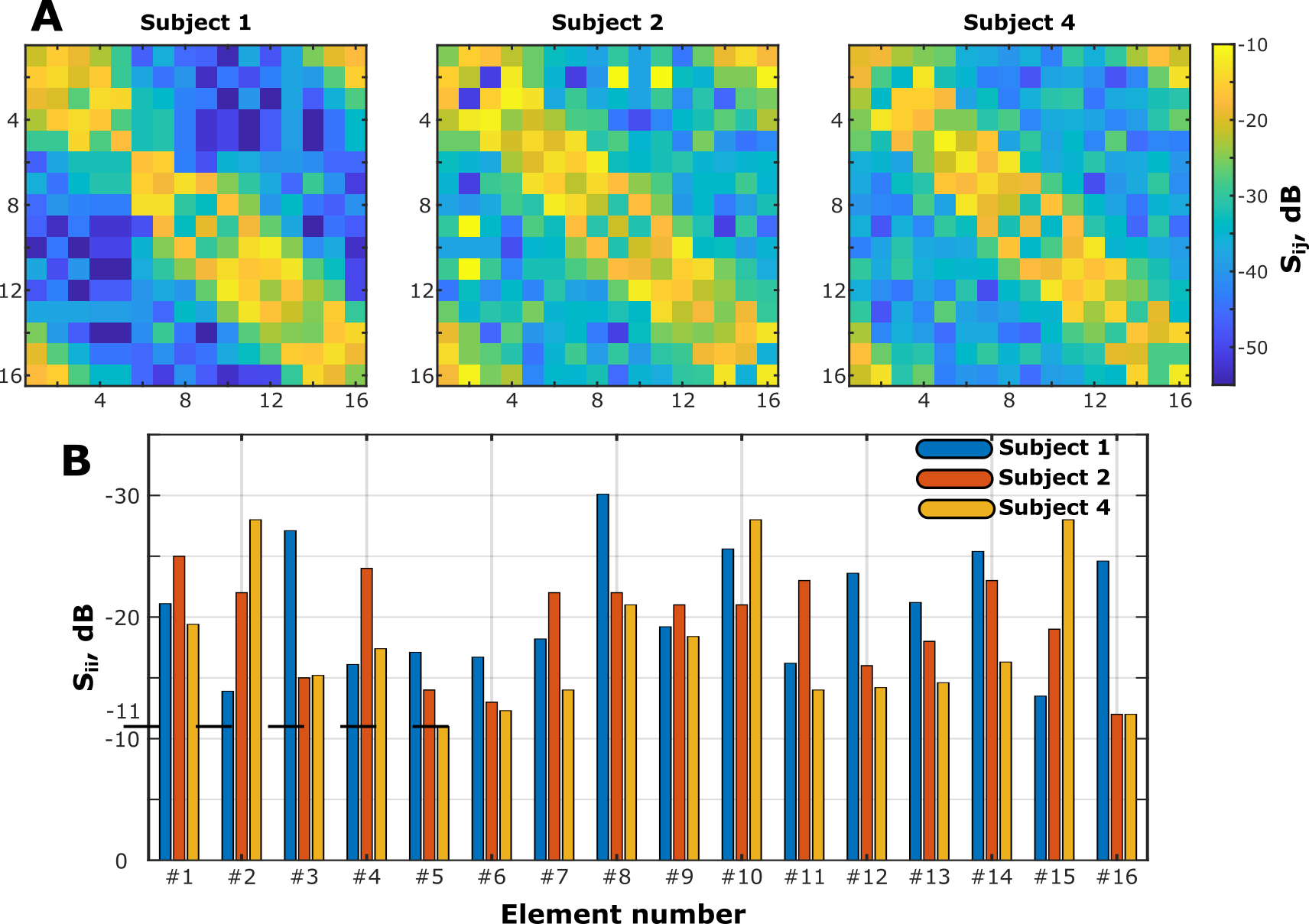

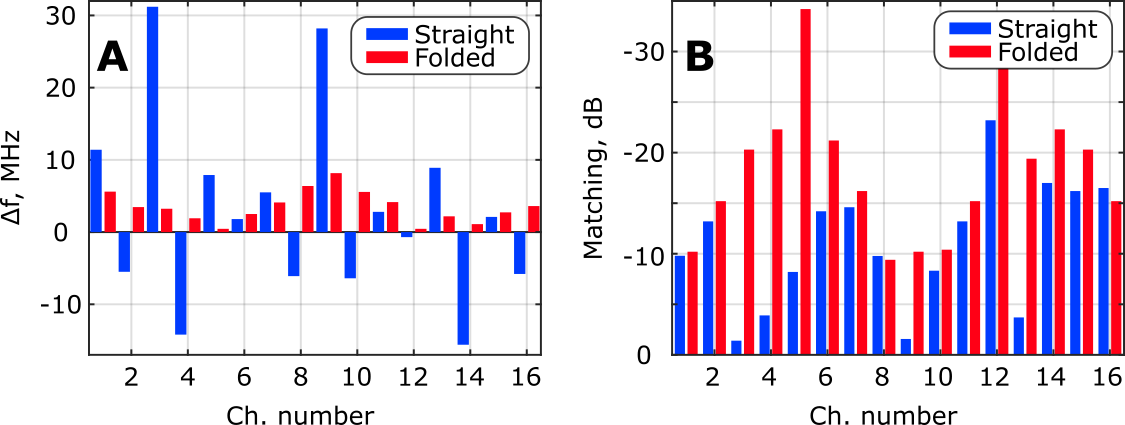


Figure S2. (A) Numerically calculated frequency shift of 20-mm gap array element input reflection coefficients when replacing the Duke with the Ella voxel model for straight and folded-end 16-channel arrays. (B) Numerically calculated matching for straight and folded-end 16-channel arrays loaded to the Ella model, but initially tuned and matched on Duke.

Figure S4. B_1_^+^ maps acquired using the vendor-provided pre-saturated TurboFLASH sequence for the three healthy volunteers displayed in a central sagittal slice.


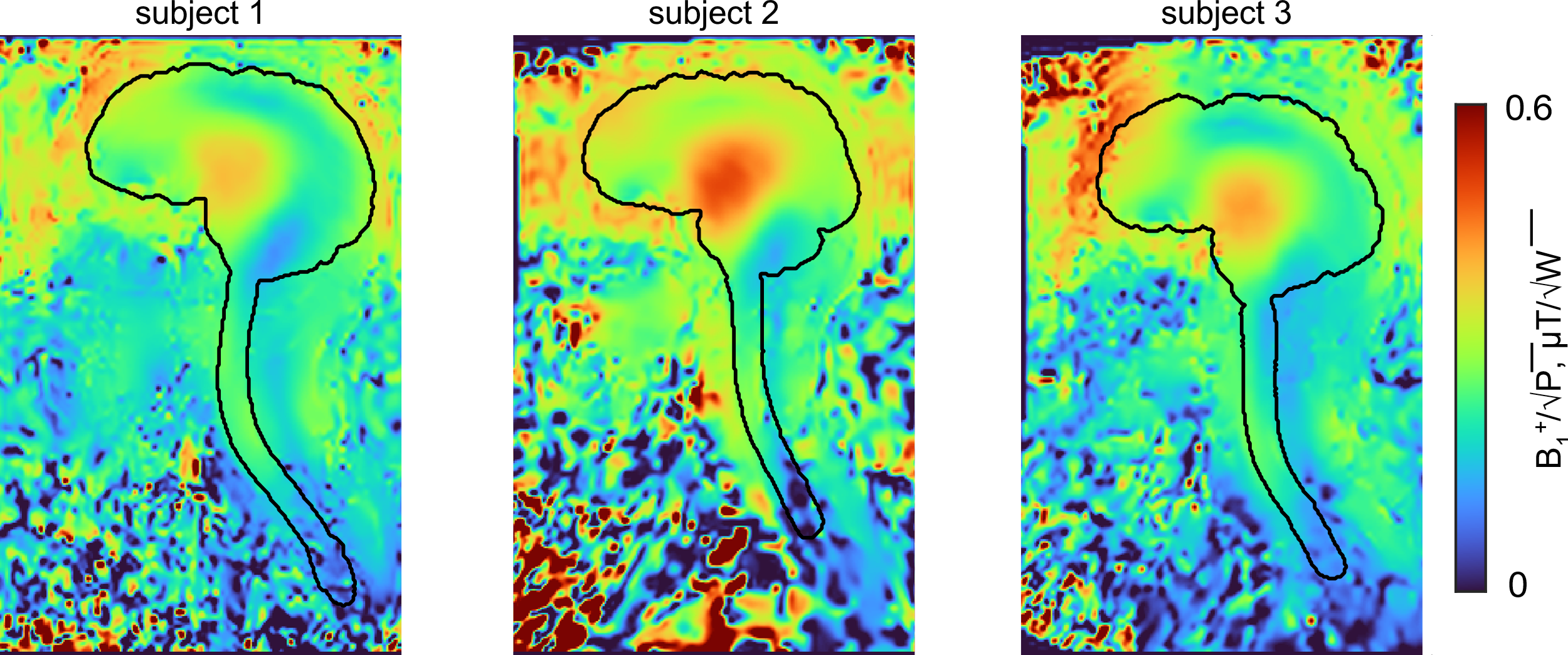

Supplement: Supplementary file 1 — Figure S1: Design of the numerical model of the 16‐channel loop array (A) and 8‐channel loop array (B) used for comparison with the optimal configuration of the dipole array (folded‐end/10‐mm extended/20‐mm gap). Transformer decoupling was implemented for decoupling between the elements in one row, and overlapping decoupling was implemented for decoupling between the rows. Figure S2: (A) Numerically calculated frequency shift of 20‐mm gap array element input reflection coefficients when replacing the Duke with the Ella voxel model for straight and folded‐end 16‐channel arrays. (B) Numerically calculated matching for straight and folded‐end 16‐channel arrays loaded to the Ella model, but initially tuned and matched on Duke. Figure S3: (A) Measured full S‐matrix at 399.72‐MHz frequency for three healthy volunteers. (B) Bar plot of Sii (i.e., matching) for different volunteers. Figure S4: B1 + maps acquired using the vendor‐provided presaturated TurboFLASH sequence for the three healthy volunteers displayed in a central sagittal slice. [file NBM-39-e70228-s001.docx]
